# Chromosomal instability and phenotypic variation in a specific lineage derived from a synthetic allotetraploid wheat

**DOI:** 10.3389/fpls.2022.981234

**Published:** 2022-08-22

**Authors:** Ruili Lv, Changyi Wang, Ruisi Wang, Xiaofei Wang, Jing Zhao, Bin Wang, Tariq Aslam, Fangpu Han, Bao Liu

**Affiliations:** ^1^School of Life Sciences, Linyi University, Linyi, China; ^2^Key Laboratory of Molecular Epigenetics of the Ministry of Education (MOE), Northeast Normal University, Changchun, China; ^3^Institute of Genetics and Developmental Biology, Chinese Academy of Sciences (CAS), Beijing, China

**Keywords:** synthetic allopolyploid, wheat, chromosomal instability, aneuploidy, structural variation, fitness

## Abstract

Newly formed plant allopolyploids usually have meiosis defect, resulting in chromosomal instability manifested as variation in chromosome number and/or structure. However, not all nascent allopolyploids are equally unstable. The wheat group (*Aegilops/Triticum*) contains 13 diploid species with distinct genome types. Many of these species can be artificially hybridized to produce viable but sterile inter-specific/intergeneric F1 hybrids, which can generate fertile synthetic allotetraploid wheats after whole genome doubling. Compared with synthetic allotetraploid wheats that contain genome combinations of AADD and S*S*DD (S* refers to related S genomes of a different species), those containing an S*S*AA genome are significantly more stable. However, robustness of the relative stability of S*S*AA genomes is unknown, nor are the phenotypic and fitness consequences during occurrences of secondary chromosomal instability. Here, we report a specific lineage originated from a single individual plant of a relatively stable synthetic allotetraploid wheat with genomes S*^l^*S*^l^*AA (S*^l^* and A subgenomes were from *Ae. longissima* and *T. urartu*, respectively) that showed a high degree of transgenerational chromosomal instability. Both numerical chromosome variation (NCV) and structural chromosome variation (SCV) occurred widely. While substantial differences in frequencies of both NCV and SCV were detected across the different chromosomes, only NCV frequencies were significantly different between the two subgenomes. We found that NCVs and SCVs occurred primarily due to perturbed meiosis, allowing formation of multivalents and univalents as well as homoeologous exchanges. Thus, the combination of NCVs and SCVs affected multiple phenotypic traits, particularly those related to reproductive fitness.

## Introduction

With a genome constitution of BBAADD, common wheat (*Triticum aestivum* L., 2*n* = 6× = 42) is a neo-allohexaploid species whose origin comprises of three diploid species. These are, respectively, *T. urartu* (AA), *Aegilops tauschii* (DD), and a yet unidentified but most probably extinct diploid species related to the five diploid species of *Aegilops*, section *Sitopsis*, which contained the S* genomes (Feldman and Levy, 2015). Recent phylogenomics-based studies (Glémin et al., 2019; Li et al., 2022) have confirmed the long suggested scenario based on morphological comparisons and circumstantial genetic evidence that the still mysterious diploid donor to the B-subgenome of polyploid wheat is most closely related to *Ae. speltoides*. This donor, however, is phylogenetically disparate from the rest of the four species of the *Sitopsis* section.

Most of the 13 diploid species of the *Triticum-Aegilops* complex can still be artificially hybridized to produce viable, though sterile, F1 hybrids, which after genome doubling can form fertile synthetic allotetraploids (Feldman et al., 1995). These newly formed allopolyploids not only provide essential materials for the study of early stages of allopolyploid evolution, but can also serve as important bridges mediating the introduction of useful genes from the diploid wild species to cultivated polyploid wheats. A salient feature of newly formed allopolyploids is perturbation of meiosis, which often results in chromosomal instability manifested as numerical and/or structural chromosome variations (Comai, 2005; Otto, 2007; Doyle et al., 2008; Gonzalo, 2022). Although it is generally believed that chromosomal instabilities are likely unavoidable byproducts of allopolyploidization due to compromised meiosis, and hence being selected against, accumulated recent evidence in unicellular organisms and human cancer cells (Sheltzer et al., 2011; Selmecki et al., 2015) has shown that both numerical and structural chromosome changes may lead to fitness leaps under certain stress conditions, such as antibiotic treatments or chemotherapies, and therefore, can represent adaptive genetic changes. Thus, the biological and evolutionary significance of chromosomal instability associated with plant allopolyploidization remains to be fully understood.

We have reported that synthetic allotetraploid wheats constructed by using various polyploid wheat-related diploid species show highly variable degrees of meiotic irregularity, and hence, chromosome instability (Zhang et al., 2013; Gou et al., 2018). Specifically, synthetic allotetraploid wheats containing S*S*AA genomes were chromosomally more stable than those containing AADD or S*S*DD genomes (Zhang et al., 2013; Gou et al., 2018). However, robustness of the relative higher stability of S*S*AA genomes is unknown, nor are the phenotypic, and especially fitness, consequences if chromosomal instability occurs in these relatively stable synthetic allotetraploid wheats.

Here, we report that during our transgenerational monitoring for chromosome stability of a synthetic wheat with a S*^l^*S*^l^*AA genome (the S*^l^* and A subgenomes were donated by *Ae. longissima* and *T. urartu*, respectively), we unexpectedly identified one lineage of a single euploid origin that showed a high degree of chromosomal instability. We investigated the pattern and trend of numerical and structural chromosome variation of this lineage over multiple generations as well as their relevance to meiotic chromosome behavior. We provide evidence that chromosomal variation has large effects on multiple phenotypes including reproductive fitness of newly formed plant allopolyploid populations. We discuss implications of our findings with respect to early stages of polyploid evolution and potential harnessing of chromosomal instability in the generation of practically useful germplasms.

## Materials and methods

### Plant materials

**FIGURE 1:**
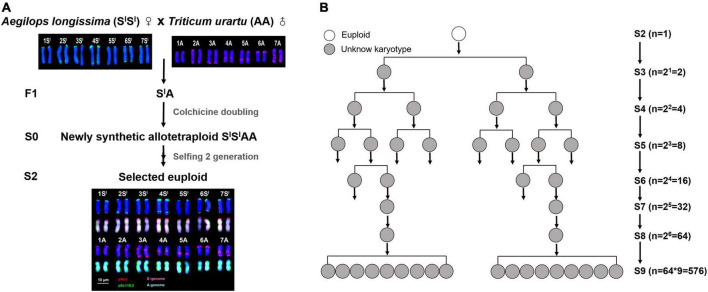
The standard karyotypes and pedigree tracing of a lineage with high degree of chromosomal instability in a synthetic allotetraploid wheat (S*^l^*S*^l^*AA). **(A)** The FISH/GISH-based standard karyotypes of S*^l^*S*^l^*AA and its diploid parents. **(B)** Pedigree of the chromosomal unstable lineage containing a total of 576 individuals at S9, which were descended from the single euploid founder plant of S2. The pAs1 (red) and pSc119.2 (green) clones were used as FISH probes. The genomic DNAs of *Ae. longissima* (S*^l^*S*^l^*, red) and *T. urartu* (AA, green) were used as GISH probes.

The plant material used in this study was a synthetic allotetraploid wheat (genome S*^l^*S*^l^*AA, 2*n* = 28), which was produced by hybridization and subsequent colchicine doubling of F1 hybrids between *Ae. longissima* (genome SS, 2*n* = 14, accession TL05) and *T. urartu* (genome AA, 2*n* = 14, accession TMU06) (Figure 1A). The original seeds (S1) of S*^l^*S*^l^*AA were provided by Professor Moshe Feldman of the Weizmann Institute of Science, Israel. One lineage that showed a high degree of chromosomal instability was identified, and pedigree traced, in which 576 individual plants at S9 were available for study (Figure 1B). Combined fluorescence *in situ* hybridization (FISH) and genomic *in situ* hybridization (GISH) (Han et al., 2004; Kato et al., 2004; Zhang et al., 2013) was used for karyotyping.

An independent pedigree was established starting from a single euploid individual of the S2 generation, in which only *bona fide* euploids were selected for propagation for seven consecutive generations of selfing (from S3 to S9). Seven euploid individuals were randomly chosen at S4 to perform FISH/GISH-based meiotic analysis, detailed below.

### Cytological identification of chromosomes in mitosis and meiosis

Mitotic metaphase cells of root-tips of all the 576 individual plants of the S9 population were karyotyped by FISH/GISH essentially according to the protocols reported (Han et al., 2004; Kato et al., 2004) with minor modifications (Zhang et al., 2013); this combinatory use of FISH/GISH allows accurate identification of each chromosome pairs of both the A- and S*^l^* subgenomes (Ruban and Badaeva, 2018). Specifically, the two cloned FISH probes, pSc119.2, a 120 bp tandem repeat cloned from *Secale cereale* (Bedbrook et al., 1980) and pAs1, a 1,000 bp tandem repeat cloned from *Aegilops tauschii* (Rayburn and Gill, 1986), were, respectively, labeled by nick translation with Alexa Fluor 488-5-dUTP (green) and Texas red-5-dCTP (red). Genomic DNA from *Ae. longissima* (S*^l^*S*^l^*) and *T. urartu* (AA) were labeled by nick translation with Alexa Fluor 488-5-dUTP and Texas red-5-dCTP, respectively. DAPI (VECTOR, H-1200) was used for chromatin counterstaining. Slides were examined under an Olympus BX53 fluorescence microscope and digitally photographed. The images were captured with camera using the IPP software.

The pollen mother cells (PMCs) at meiotic metaphase I and anaphase I of seven *bona fide* euploid plants at S4 were sampled for meiosis analysis (Armstrong, 2013). Young inflorescences (*ca.* 3–4 cm) at desired stage were fixed in Carnoy’s solution (ethanol: acetic acid = 3: 1), and anthers were dissected to spread meiocytes at meiotic metaphase I and anaphase I. Meiotic chromosome pairing and segregation behaviors were tabulated and quantified. Meiotic FISH method and probes (pSc119.2 and pAs1) were the same as used for mitotic karyotyping, described above. The images were collected as for those of mitosis, described above.

### Phenotyping

Seedlings of all 576 individuals of the S9 population were placed in the same phytotron, at 25°C for 16 h light in the daytime and 18°C for 8 h darkness at night to grow to the 3rd-leaf stage. The seedlings were then transferred to a 4°C incubator for 30 days of vernalization. All plants were transplanted to the experimental field and grew to maturity. Six phenotypic traits including plant height, spike length, spikelet density, seed setting, grain length and grain width, which reflect both growth/development and reproductive fitness were quantified (Gou et al., 2018).

### Statistics

Statistical significance of respective comparisons and graphical illustration were executed in R (v.3.2.2) (R Core Team, 2012) and EXCEL. *P*-value < 0.05 as cut off in all statistic test. Specifically, Binomial test was used to (i) compare the predisposition of NCV between subgenomes and among chromosomes; (ii) compare the predisposition of SCV between subgenomes; and (iii) compare the frequencies of early disjunction between subgenomes. Chi-square test was used to (i) compare the predisposition of SCV among chromosomes; and (ii) compare the frequencies of NCVs and/or SCVs between selected euploid plants and non-karyotyped plants to produce the next generation. Least significant difference (LSD) test was used to compare the phenotypic difference among four karyotype groups.

## Results

### Persistent karyotype instability occurred in a specific lineage from a relatively stable synthetic allotetraploid wheat (genome S*^l^*S*^l^*AA)

A synthetic allotetraploid wheat (genome S*^l^*S*^l^*AA, 2*n* = 28), was produced by inter-genetic hybridization followed by colchicine doubling of F1 hybrids between two diploid species, *Aegilops longissima* (genome S*^l^*S*^l^*, 2*n* = 14) and *Triticum urartu* (genome AA, 2*n* = 14) (Figure 1A). This synthetic allotetraploid wheat was chromosomally much more stable compared with those with other genome combinations, such as AADD and S*S*DD (Zhang et al., 2013). However, during our transgenerational monitoring for chromosome stability of randomly chosen individuals of this synthetic wheat, we unexpectedly identified one lineage that showed a high degree of chromosomal instability. We traced the pedigree of this lineage and identified it as originating from a single euploid individual at the second selfed generation (S2). We found this lineage contained a total of 576 individual plants that were descended from this single S2 founder plant by S9, the pedigree of which was clarified as two daughter individuals from one mother plant at each of the intervening S3 to S8 generations (Figure 1B). We thus analyzed all these 576 plants of S9 by using sequential fluorescence *in situ* hybridization (FISH) and genomic *in situ* hybridization (GISH) (Han et al., 2004; Kato et al., 2004; Zhang et al., 2013) that enables accurate identification of each of the 14 chromosome pairs of this synthetic allotetraploid wheat (Ruban and Badaeva, 2018). We obtained clear karyotypes for 526 individual plants.

**FIGURE 2:**
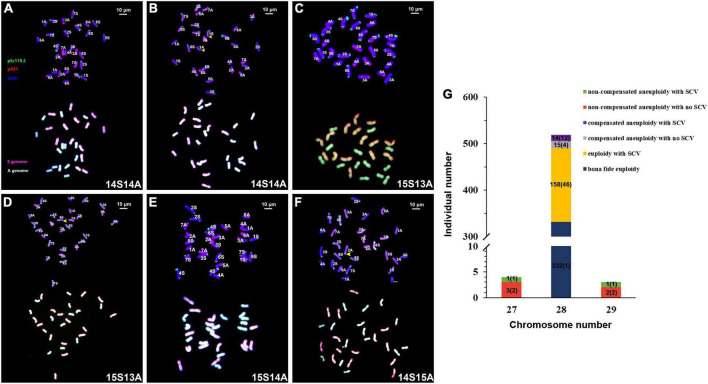
Illustration of the six karyotypic categories, the distribution of relative proportions of plants belonging to each of the categories, and the numbers of distinct karyotypes contained by each category among the 526 karyotyped individuals at S9 of the specific lineage of synthetic allotetraploid wheat (S*^l^*S*^l^*AA). Images of are illustrations of the six karyotypic categories, *bona fide* euploid **(A)**, euploid with SCVs **(B)**, compensated aneuploid with no SCVs **(C)**, compensated aneuploid with SCVs **(D)**, non-compensated aneuploid with no SCVs **(E)** and non-compensated aneuploid with SCVs **(F)**. The pAs1 (red) and pSc119.2 (green) are used as FISH probes. Yellow arrows refer to SCVs. The genomic DNAs of *Ae. longissima* (S*^l^*S*^l^*, red) and *T. urartu* (AA, green) were used as GISH probes. The bar-chart **(G)** is distribution of relative proportions of plants belonging to each of the categories, while the numbers in parenthesis numbers of distinct karyotypes identified in each category.

The 526 individual plants could be categorized into six groups according to their karyotypes constituted by the absence or presence of numerical chromosome variations (NCVs) and/or structural chromosome variations (SCVs), as defined previously (Gou et al., 2018). Group 1 was “*bona fide* euploidy,” i.e., the karyotype of each subgenome was the same as that of its diploid parental donor species (2*n* = 28 = 14S*^l^* + 14A); there were 332 individual plants in group 1, accounting for 63.1% of the total 526 individual plants (Figures 2A,G). Group 2 was “euploidy with SCV,” i.e., each subgenome contained 14 chromosomes (2*n* = 28 = 14S*^l^* + 14A), but at least one chromosome containing one SCV; there were 158 individuals in group 2, accounting for 30.0% of the total 526 plants (Figures 2B,G and Supplementary Table 1). Group 3 was “compensated aneuploidy with no SCV,” i.e., the total chromosome number was 28, but at least one chromosome was deviated from the normal two copies, showing a loss/gain of one or more homoeologous chromosome pairs (2*n* = 28, S*^l^*≠14, A≠14), in which no chromosome contained SCV; there were 15 individuals in group 3, accounting for 2.9% of the total 526 individuals (Figures 2C,G and Supplementary Table 1). Group 4 was “compensated aneuploidy with SCV,” i.e., the same as in group 3 but at least one chromosome contained SCV; there were 14 individuals in group 4, accounting for 2.7% of total 526 individuals (Figures 2D,G and Supplementary Table 1). Group 5 was “non-compensated aneuploidy with no SCV,” i.e., the chromosomes number was not 28 (2*n* ≠ 28), and no chromosome contained SCV; there were 5 individuals in group 5, accounting for 1% of the 526 individuals (Figures 2E,G and Supplementary Table 1). Group 6 was “non-compensated aneuploidy with SCV,” the same as group 5, but at least one chromosome contained SCV; there were only two individuals in group 6, accounting for 0.4% of the total 526 individuals (Figures 2F,G and Supplementary Table 1). Notably, apart from the 321 plants of group 1, i.e., *bona fide* euploidy, which contained a single karyotype, all the rest five groups contained multiple karyotypes, producing a total of 69 distinct karyotypes (Figure 2G). This karyotype diversity was mainly conditioned by SCVs, as NCVs were limited to loss or gain of one chromosome only, i.e., 2*n* = 27 or 29 (Figure 2G).

### Frequencies of numerical chromosome variation differed both among chromosomes and between subgenomes

Of the 526 individual plants, 36 (2 + 5 + 14 + 15 = 36) showed NCVs, i.e., aneuploidies (Figure 2G). We thus asked two questions: First, were the 14 chromosome pairs (seven of each of the S*^l^* and A subgenomes) equal in their probabilities to be in an aneuploid state? Second, were the probabilities of chromosomal gain vs. loss equal?

**FIGURE 3:**
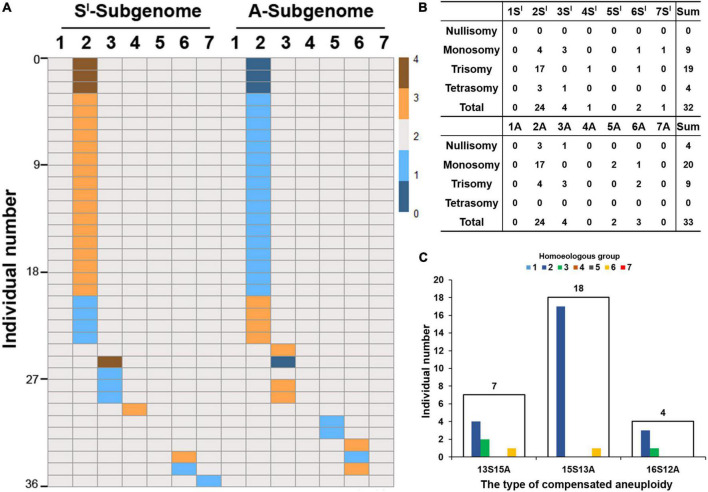
Numerical chromosome variations (NCVs) in the 526 karyotyped individuals. The heatmap **(A)** shows gain or loss of chromosomes among the 14 chromosome pairs of subgenomes S*^l^* and A, which occurred in 36 of the 526 individuals. Color key represents chromosome copies from 0 to 4. **(B)** Is distribution of the numbers of individuals showing variable copies (from 0 to 4) of each of the 14 pairs of chromosomes. **(C)** Depicts the numbers of plants in the three different types of compensated aneuploidies. The digits above the frames are numbers of individuals belonging to each of the three types of compensated aneuploidies. The colored bars are individual numbers belonging to each of the seven homoeologous groups within each of the three types of compensated aneuploidies.

We found that the 14 chromosome pairs varied greatly in their probabilities to be in an aneuploid state or to show loss vs. gain of a chromosome. First, five chromosome pairs, i.e., those of 1S*^l^*, 5S*^l^*, 1A, 4A, and 7A, did not show any NCV, while the rest of the nine showed NCVs (Figures 3A,B). Second, of the five chromosome pairs of S*^l^*-subgenome, gain and loss were not found in all instances and did not occur in equal frequencies. Specifically, only four chromosome pairs (2S*^l^*, 3S*^l^*, 4S*^l^*, and 6S*^l^*) showed gain of chromosome(s), with 2S*^l^* showing a staggeringly higher frequency (87%) than the other three chromosome pairs, which were at an equal frequency (4.3%) (Binomial test, *P* = 2.42E-15) (Figures 3A,B); only chromosome pairs 2S*^l^*, 3S*^l^*, 6S*^l^*, and 7S*^l^* were more prone to the loss of a chromosome, and of which 2S*^l^* and 3S*^l^* showed higher frequencies (44.4 and 33.3%, respectively) than did 6S*^l^* and 7S*^l^* (both with a frequency of 11.1%) (Binomial test, *P* = 6.26E-04) (Figures 3A,B). Third, of the seven chromosome pairs in A-subgenome, only those of 2A, 3A, and 6A showed gain of a chromosome, at frequencies of 44.4, 33.3, and 22.2%, respectively (Figures 3A,B). Likewise, only chromosomes 2A, 3A, 5A, and 6A showed loss at frequencies of 83.3, 4.2, 8.3, and 4.2%, respectively (Figures 3A,B). Third, chromosomes 1S*^l^*/1A showed neither gain nor loss of a chromosome (Figures 3A,B). Finally, when considering each subgenome as a whole, subgenome S*^l^* was more likely to gain chromosomes than subgenome A (Binomial test, *P* = 1.95E-07), and subgenome A was more likely to lose chromosomes than subgenome S*^l^* (Binomial test, *P* = 1.95E-07) (Figures 3A,B).

We further analyzed the compensated aneuploidy, i.e., the categorized groups 3 and 4 plants, described above, in detail, which included a total of 29 individuals (15 in group 3 + 14 in group 4) (Figure 2G). We found that 22 individuals (75.9%) harbored ≥15 S*^l^*-subgenome chromosomes, while only seven individuals (24.1%) contained ≤13 S*^l^*-subgenome chromosomes (Figure 3C). The frequency of S*^l^*-subgenome chromosome gain/A-subgenome chromosome loss was significantly higher than the opposite trend (Binomial test, *P* = 6.55E-09). Thus, the trend of differential gain vs. loss exhibited by chromosomes of the two subgenomes for plants of the compensated aneuploidy groups was the same as in the whole plant population, described above. Taken together, it is clear that the frequencies of NCV varied both among chromosomes and between the two subgenomes in this specific lineage of the synthetic allotetraploid wheat with genomes S*^l^*S*^l^*AA.

### Frequencies of structural chromosome variation differed among chromosomes but which did not cumulate to differences between subgenomes

**FIGURE 4:**
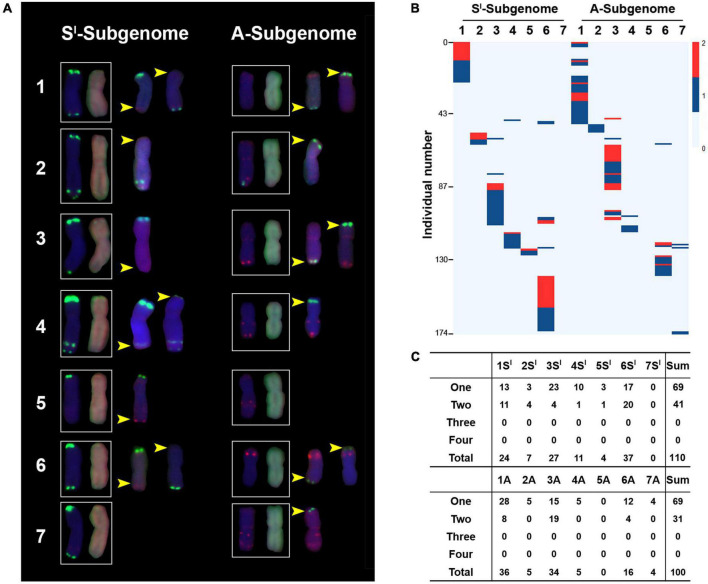
Structural chromosome variations (SCVs) in the 526 karyotyped individuals. Illustration of representative SCVs occurred to each of the 14 chromosome pairs **(A)**. The pAs1 (red) and pSc119.2 (green) were used as FISH probes. The genomic DNAs of *Ae. longissima* (S*^l^*S*^l^*, red) and *T. urartu* (AA, green) were used as GISH probes. Yellow arrows denote SCVs. The numbers of individuals containing SCVs occurred to each of the 14 chromosome pairs of S*^l^* and A subgenomes, which occurred in a total of 174 individuals **(B)**. Color key represents with SCVs, which varied from 0 to 2. **(C)** Detailed numbers of individuals with chromosome copies containing SCVs in each of the 14 chromosome pairs. “One to Four” refer to chromosome copies.

The combined FISH/GISH enabled the identification of microscopically discernible SCVs in this synthetic allotetraploid wheat at high resolution (S*^l^*S*^l^*AA) (Figure 4A). We found several features of the SCVs, including (i) all SCVs occurred at subtelomeric regions; (ii) the SCVs were highly similar in both position and size between a given homoeologous chromosome pair in the allotetraploid wheat S*^l^*S*^l^*AA; (iii) all clearly discernible SCVs were homoeologous exchanges (HEs); and (iv) most of the SCVs were manifested as non-reciprocal (Figure 4A). These features suggest that the major mechanism for the occurrence of SCVs in this synthetic allotetraploid wheat was meiotic homoeologous exchanges (HEs), while the often non-reciprocal manifestation should be due to meiotic chromosome segregation and/or gamete/sporophyte selection rather than occurrence.

A total of 174 (2 + 14 + 158 = 174) individuals were found to have SCVs in the 526 karyotyped plants, accounting for 33.0% (Figure 2G). The 14 chromosome pairs showed highly variable frequencies of SCV (Figure 4B). Specifically, (i) among the seven chromosome pairs of the S*^l^*-subgenome, no SCV was detected in 7S*^l^*, and SCVs in 1S*^l^*, 3S*^l^*, and 6S*^l^* were 21.8, 24.5, and 33.6%, respectively, while those in 2S*^l^*, 4S*^l^*, and 5S*^l^* were 6.4, 10.0, and 3.6%, respectively, rendering frequencies of SCVs in the former three chromosome pairs being significantly higher than those in the later three chromosome pairs (Chi-square test, *P* = 1.59E-13) (Figures 4B,C); (ii) among the seven chromosome pairs of the A-subgenome, no SCV was detected in 5A, and SCVs in 1A, 3A, and 6A were 36.0, 34.0, and 16.0%, respectively, while those in 2A, 4A, and 7A were 5.0, 5.0, and 4.0%, respectively, rendering frequencies of SCVs in the former three chromosome pairs being significantly higher than those in the later three chromosome pairs (Chi-square test, *P* = 4.10E-18) (Figures 4B,C). In theory, there could be three or four SCVs for a given homoeologous chromosome group in cases of monosomy/trisomy and nullisomy/tetrasomy, respectively. Nevertheless, we did not observe such situations (Figures 4B,C). Taken together, it is clear that homoeologous chromosome pairs tended to show highly similar tendencies of SCVs, confirming that HE was the major mechanistic underpinning its formation. Because of similar frequencies in SCVs between homoeologous chromosome pairs, when each subgenome was considered as a whole, no significant difference was detected between the S*^l^* and A subgenomes (Binomial test, *P* = 5.35E-01), as expected (Figures 4B,C).

### Persistent chromosomal instability was not due to cascading effect of a driver numerical or structural chromosomal variation

**FIGURE 5:**
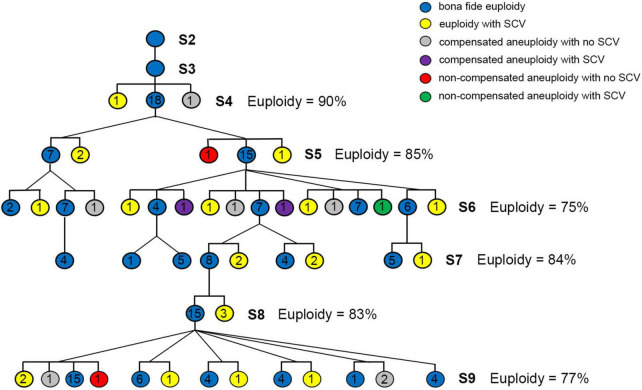
Construction of an independent pedigree starting from a randomly chosen S3 plant derived from the same seeding S2 plant that gave rise to the chromosomal unstable linkage described in Figure 1, and karyotype analysis. The pedigree consisted of seven consecutive generations of selfing by selecting one random euploid individual at each of the intervening generation (S3–S9) to produce the next generation. From 18 to 44 individual plants were randomly chosen for karyotyping in each generation to calculate the frequencies of euploidy. The six colored circles represent the six karyotypic categories, and the numbers in the circles represent the number of individuals contained in each category.

As said above, we have traced the origin of the plant lineage that showed chromosomal instabilities to a specific S2 euploid individual and tracked the propagation model at each generation, i.e., all being two daughter individuals from a preceding mother plant (Figure 1B). However, because we did not analyze the intervening generations (from S3 to S8), we had no information about the karyotypes of these individuals (Figure 1B). The result that 332 (63.1%) of the 526 karyotyped individuals of S9 were “*bona fide* euploidy” may strongly suggest that most contributing plants at the non-karyotyped intervening generations were likely euploidy, this however could not be ascertained because in theory any plant that did not contain NCVs and SCVs at a homozygous state could produce *bona fide* euploid progenies. Given findings in yeast and human cancer cells that a driver chromosomal variant (especially aneuploidy) may catalyze additional NCVs and SCVs in a cascading manner (Sheltzer et al., 2011), it was interesting to test if this could be the case in this specific lineage with chromosomal instability of the synthetic allotetraploid wheat (S*^l^*S*^l^* AA) that was otherwise highly stable (Zhang et al., 2013). To test this possibility, we conducted GISH/FISH analysis of S4 seeds derived from one S3 euploid that was from the same S2 euploid founder plant (Figure 1B), and randomly chose a *bona fide* euploid seed to construct an independent pedigree also including seven consecutive generations of selfing (from S3 to S9), in which we conducted karyotyping for variable numbers of randomly chosen plants at each generation, and only selected euploid plants to produce the next generation (Figure 5). We found that from S5 to S9, the proportions of *bona fide* euploid plants were in the range of 75–85%, leaving the collective proportions of plants with NCVs and/or SCVs in the range of 15–25% (Figure 5). These frequencies were lower than that of the 526 plants derived from non-karyotyped plants at the intervening generations, which was 36.9% (1–0.631, Chi-square test, *P* = 0.03) (Figure 1B). This however was within expectation, as any NCV- and/or SCV-containing individuals would produce more progenies containing the same NCV and/or SCV. If such a plant was homozygous for the NCV/SCV, then all its progenies would contain the variant. Furthermore, there was no increases in the *bona fide* euploidy proportions with generation progression (Figure 5). Together, these results suggest that the transgenerationally persistent chromosomal instabilities of this specific lineage of the synthetic allotetraploid wheat (S*^l^*S*^l^* AA) was not cascaded by a driver NCV or SCV, but more likely due to genetic/epigenetic mutation of a critical gene(s) involved in the meiosis machinery.

### Both numerical chromosome variations and structural chromosome variations were causally linked to perturbed meiosis

**FIGURE 6:**
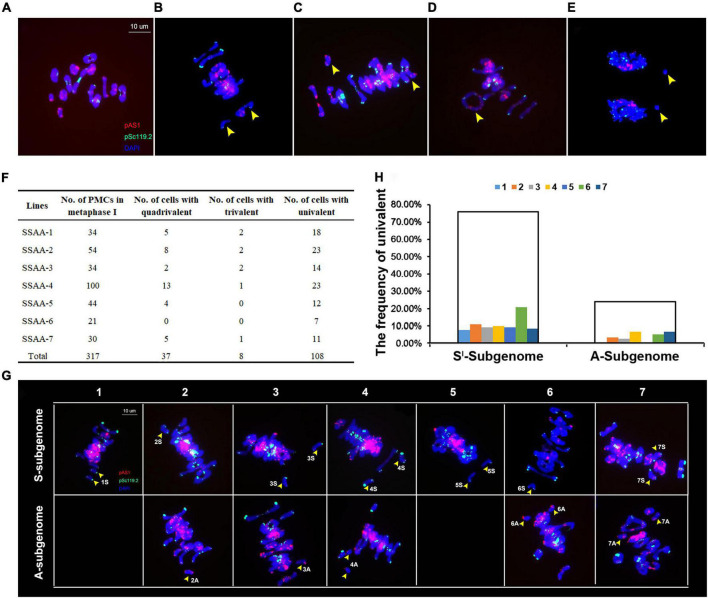
Cytological chromosome behavior at metaphase and anaphase of meiosis I of pollen mother cells (PMCs) taken from seven *bona fide* euploid individuals randomly chosen at S4. **(A)** Normal chromosome pairing at metaphase I, in which 14 bivalents were formed *via* exclusive homologous chromosome pairing. **(B)** Two univalents at metaphase I. **(C)** A univalent and a trivalent at metaphase I. **(D)** A quadrivalent at metaphase I. **(E)** Lagging univalents at anaphase I. The FISH images were probed by pAs1 (red) and pSc119.2 (green), and counterstained by DAPI. Yellow arrows denote abnormal chromosome pairing (univalent or multivalents) and disjunction. **(F)** Tabulated PMCs showing chromosome pairing behavior at metaphase I. **(G)** FISH images illustrate univalents (yellow arrows) of each of the 14 chromosome pairs of both S*^l^* and A subgenomes at metaphase I. The pAs1 (red) and pSc119.2 (green) were used as FISH probes. **(H)** The frequencies each of the 14 chromosome pairs of both S*^l^* and A subgenomes showing univalents.

The foregoing results suggested that both NCVs and SCVs were most probably due to perturbed meiosis in the founder S2 euploid individual of the synthetic allotetraploid wheat. Because the original S2 individual was not available for study, we randomly chosen seven euploid individuals at S4 derived from the same S2 founder plant, and performed FISH-based meiotic analysis (Figures 6A–E). In total, 317 pollen mother cells (PMCs) at metaphase of meiosis I (MI) were examined and chromosome pairing behavior quantified (Figure 6F). Results showed that 181 (57.1%) of the 317 PMCs at MI showed bivalent formation (ring- or rod-shaped), while the rest 136 (42.9%) MI cells contained one or more types of abnormal pairing including quadrivalent (37 PMCs) and trivalent (8 PMCs), or no pairing, i.e., univalent (108 PMCs) (Figure 6F). Of the 37 quadrivalents detected, 29 (78.38%), 4 (10.81%) and 5 (13.51%) were formed by chromosomes 2S*^l^*-2A, 3S*^l^*-3A, and 6S*^l^*-6A, respectively (Supplementary Figure 1), consistent with the staggeringly higher frequencies of NCVs of chromosomes 2S*^l^* and 2A (Figure 3). In addition, certain proportions of bivalents (presumably the rod-shaped ones) as well as multivalents (quadrivalents and trivalents) showed early disjunction (Figure 6G). The frequencies of early disjunction were variable both across the chromosome pairs and between the two subgenomes (Figures 6G,H). Across the 14 chromosome pairs, 6S*^l^* showed the highest frequency (20.83%) of early disjunction, while between the two subgenomes, the frequency of early chromosome disjunction was significantly higher in the S*^l^*-subgenome (75.83%) than in the A-subgenome (24.17%) (Binomial test, *P* = 8.63E-33) (Figure 6H), consistent with more NCVs in S*^l^*- than in A-subgenome (Figure 3). These aberrant chromosome pairing behaviors at MI resulted in unbalanced chromosome segregations at anaphase of meiosis I (AI). Of a total of 105 AI PMCs examined, 26 (24.8%) cells contained lagging chromosomes (Supplementary Table 2). Together, these results indicate that both NCVs and SCVs were due to perturbed meiosis in this specific lineage of the synthetic allotetraploid wheat with a S*^l^*S*^l^*AA genome combination.

### Phenotypic consequences of karyotypic variation at the population level

**FIGURE 7:**
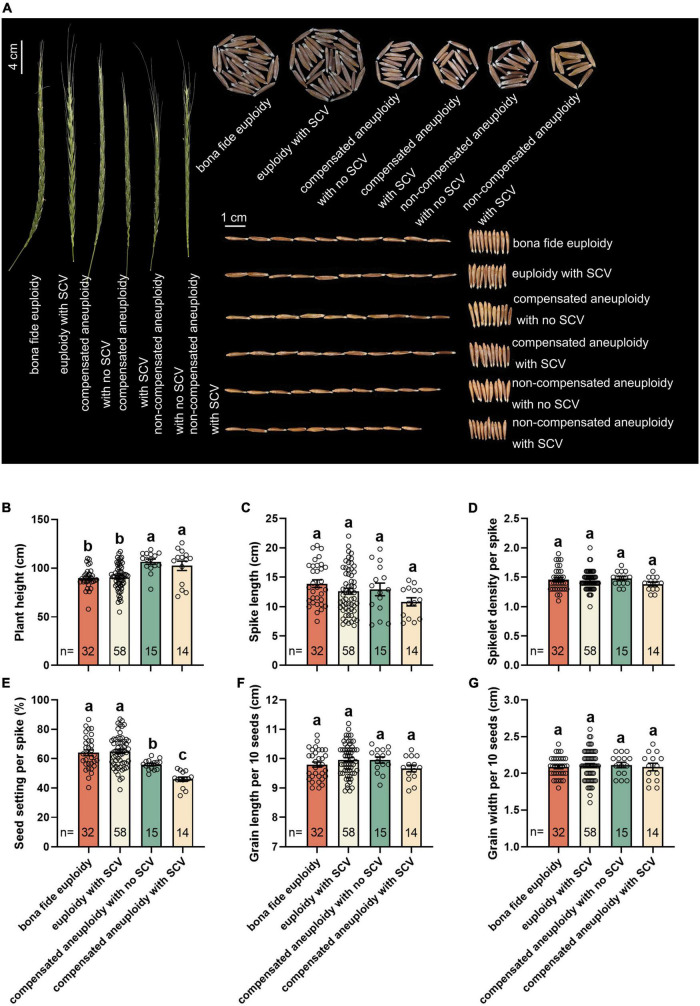
Variation in phenotypic traits across the phenotypic categories. **(A)** Variation in spike, seed-setting/individual, seed-length and seed-width among the six karyotypic categories (Figure 2). Quantification of the six phenotypic traits, plant height **(B)**, spike length **(C)**, spikelet density per spike **(D)**, seed setting per spike **(E)**, grain length per 10 seeds **(F)**, and grain width per 10 seeds **(G)**, among four karyotypic categories that each had sufficient number of plants enabling the analysis. The *y*-axis is mean (± SD). Different letters on the bars indicate significant differences based on the least significant difference (LSD) test (*P* < 0.05). The numbers (n) inside the bars refer to individual numbers in each of the four karyotypic categories.

The combination of transgenerational NCVs and SCVs generated a diverse set of distinct karyotypes by the S9 population of this specific lineage of the synthetic allotetraploid wheat (S*^l^*S*^l^*AA). Of the 526 randomly karyotyped plants of S9, 69 different karyotypes were identified (Figure 2G). To assess the phenotypic consequences of karyotype variation, we measured six traits related to both vegetative and reproductive growth and development, including plant height, spike length, spikelet density, seed setting, grain length and grain width (Figure 7). Variation in some, but not all, of these traits were evident within this small S9 population. To test for possible correlations between karyotype and trait variations, each trait was quantified as mean and variance within each of the six plant groups categorized according to karyotypes (Figure 2). Due to insufficient numbers of plants of the two categories, “non-compensated aneuploidy with no SCVs” and “non-compensated aneuploidy with SCVs” for statistical analysis, they were not included in the quantitative comparisons. Notably, however, by visual examination, these two karyotypic groups apparently showed the largest variation from the euploidy control and the other karyotypic groups in at least four of the six traits (Figure 7A). The rest of the four karyotypic groups were subjected to quantitative comparisons for each of the six traits. Results showed that significant differences existed between the karyotypic groups for two traits, plant height and seed-setting (Figures 7B,E). While the rest of the four traits, spike length, spikelet density, grain length and grain width, did not show statistical difference between the groups (Figures 7C,D,F,G). Interestingly, the two compensated aneuploidies (with and without SCVs, respectively) grew significantly taller than the two groups of euploids (with and without SCVs, respectively), whereas the opposite trend was observed for seed-setting (Figures 7B,E). Moreover, the two groups of euploids also differed in seed-setting, plants with SCVs produced more seeds than that without SCVs (Figure 7E).

## Discussion

Polyploidy entails either an abrupt whole genome doubling (WGD) alone (autopolyploidy) or WGD together with merging of divergent genomes of different species into a common nucleus (allopolyploidy). This macro-mutation jeopardizes the fine-tuned meiosis machinery and compromises chromosome pairing fidelity and segregation precision (Grandont et al., 2013; Morgan et al., 2021; Gonzalo, 2022). As a result, chromosomal instability would inevitably occur in sexually propagated progenies of newly formed polyploids, which may incur fitness penalties. Conceivably, if chromosomal instability is too severe or transgenerationally persistent, the newly formed polyploids will rapidly perish. Yet, the fact that polyploidy presents in the evolutionary histories of all higher plants (Otto and Whitton, 2000; Jiao et al., 2011; Wendel, 2015; Soltis and Soltis, 2016; Van De Peer et al., 2017) suggests that meiosis disruption at the early stages of polyploidy can be resolved in certain circumstances.

As excellently summarized by a recent review (Gonzalo, 2022), there could be multiple routes whereby meiosis instability can be surmounted in newly formed polyploids, and which conceivably are mechanistically different between auto- and allopolyploidy (Grandont et al., 2013; Morgan et al., 2021). For allopolyploid species of the *Triticum-Aegilops* complex, it was proposed that the diploid-like meiotic behavior might involve two independent mechanisms which may act additively or synergistically (Feldman et al., 1997; Ozkan et al., 2001). According to this hypothesis, one mechanism involves genetic differentiation between homoeologous chromosomes (due to parental legacy and/or post-polyploidy rapid divergent evolution of subgenomes), and the other is the presence (inherited from a diploid parent) and/or neo-functionalization of genes restricting meiotic pairing between homoeologous chromosomes, such as the *Ph1* (Okamoto, 1957; Riley and Chapman, 1958; Sears and Okamoto, 1958; Riley, 1960; Rey et al., 2018), *Ph2* (Mello-Sampayo, 1971; Serra et al., 2021), and *Ph3* (Driscoll, 1972; Mello-Sampayo and Canas, 1973) genes identified in polyploid wheat. In wheat, while the *Ph1* and *Ph2* genes are known to be located on chromosomes 5B and 3D, respectively, *Ph3* was mapped to the short-arm of chromosome 3A. The synthetic allotetraploid wheat S*^l^*S*^l^*AA apparently does not contain the *Ph1* and *Ph2* genes but might contain *Ph3* (assuming no variation among accessions of *T. urartu* or the accession harboring the genes has been used to construct the allotetraploid wheat). Thus, it is possible that *Ph3* plays a role in S*^l^*S*^l^*AA rendering its meiosis significantly more stable than that of other synthetic allotetraploid wheats such as those of AADD and S*S*DD (Zhang et al., 2013). This hypothesis, however, is yet to be confirmed with follow-up experiments.

As mentioned earlier, our prior studies indicate that synthetic allotetraploid wheats containing S*S*AA genomes were chromosomally more stable than those containing AADD or S*S*DD genomes (Zhang et al., 2013; Gou et al., 2018). Nevertheless, whether the relative chromosomal stability of synthetic wheats with S*S*AA genomes may go astray during sexual propagation and results in secondary chromosomal inability remains unknown, nor are the phenotypic (in particular fitness) consequences if chromosomal instability indeed occurs. These issues bear important evolutionary implications. For example, if chromosomal instability occurs and if progenies with NCVs and/or SCVs are transitorily (in evolutionary sense) fitter and possess higher fecundity than their euploid siblings, then the whole population will be replaced by aneuploid individuals intermingled with structural variants after a certain number of generations, which in the long run is unlikely to establish in nature.

We report here that the relative chromosomal stability of a synthetic allotetraploid wheat with genomes S*^l^*S*^l^*AA is not robust. Specifically, we incidentally identified a lineage with a single euploid origin, which shows transgenerational chromosomal instability resulting in both NCVs and SCVs. We demonstrate that both NCVs and SCVs occur widely but vary significantly among chromosomes and/or between subgenomes. To a large extent, disturbed meiotic chromosome pairing, including formation of multivalents and univalents, homoeologous exchanges (Gaeta and Chris Pires, 2010; Lloyd et al., 2018; Mason and Wendel, 2020; Wu et al., 2021), and early chromosome disjunction during anaphase (Birchler et al., 2009), can adequately explain both NCVs and SCVs in the progenies. However, differential compensating capacities between homoeologous chromosomes during both gametal and/or saprophytic development may also play a role, explaining incongruences in some homoeologous chromosome pairs between the degrees of meiosis abnormality and extents of NCVs and SCVs in the progenies. Notably, with respect to NCVs, chromosomes of the S*^l^* subgenome showed a propensity to gain additional copies while those of the A subgenome tended to lose copies. This might be due to the presence of gametocidal genes in the S*^l^* subgenome of *Ae. longissima* (Endo, 1982), which act as meiotic drivers causing preferential retention of chromosome bearing these genes (Endo, 2007; Niranjana, 2017); this however may remain cryptic in “normal” individuals whose meiosis is relatively stable (Zhang et al., 2013) but manifests the effects in this specific lineage whose meiosis stability was impaired. With respect to the differential propensities by the different chromosomes to show SCVs, since they were under the same nuclear environment, it is conceivable that differences in overall genetic similarity and/or proportion/distribution of collinearity across the seven homoeologous chromosome groups might be the underlying cause.

In this study, we also constructed and analyzed an independent pedigree in which only euploid plants were selected to produce the next generation, and plants from each generation were sampled for karyotyping. Results from these analyses provide strong circumstantial evidence that the transgenerational chromosomal instability of this lineage was not caused by a seeding NCV or SCV, but more likely due to a heritable genetic or epigenetic mutation of a key gene involved in the meiosis machinery. In this aspect, it is reminiscent to several earlier studies that have documented that this synthetic allotetraploid wheat manifested rapid genomic changes at the molecular level, including non-random elimination of low-coy DNA sequences (Ozkan et al., 2001), an overall reduction of DNA content (Ozkan et al., 2003), and gain and loss of DNA markers presumably due to nucleotide sequence mutation (Shcherban et al., 2007). This is consistent with findings in human cancer cells showing a causal relationship between chromosomal instability and molecular level genomic changes (Sheltzer et al., 2011). With respect to morphology, we show that both NCVs and SCVs have phenotypic consequences. Of note, in relation to reproductive fitness (i.e., seed-setting), the effects of NCVs are generally negative. In contrast, SCVs, especially occurring alone (i.e., in euploidy) can have positive effects. This suggests that induced homoeologous exchanges (HEs) might be practically useful in polyploid wheat breeding, as they were in other neoallopolyploid crops such as those in *Brassica* (Pires et al., 2004; Lloyd et al., 2018), *Musa* (Wang et al., 2019), *Fragaria* (Edger et al., 2019), and *Arachis* (Bertioli et al., 2019). This realization might be of special significance given that contemporary wheat cultivars hardly contain HEs due to effects of the homoeologous pairing control genes, such as *Ph1*, *Ph2*, and *Ph3*.

## Data availability statement

The original contributions presented in the study are included in the article/Supplementary material, further inquiries can be directed to the corresponding authors.

## Author contributions

BL and FH: conceptualization, methodology, and writing – review and editing. RL: data curation. RL and CW: formal analysis and writing – original draft. RL, CW, RW, XW, JZ, BW, and TA: investigation. All authors contributed to the article and approved the submitted version.

## Supplementary material

Supplementary Figure 1 Occurrence and distribution frequencies of quadrivalents at metaphase I of pollen mother cells (PMCs) among three homoeologous chromosome pairs in which quadrivalent occurred. These were 2S*^l^*-2A **(A)**, 3S*^l^*-3A **(B)**, and 6S*^l^*-6A **(C)**; their distributions were quantified **(D)**. The pAs1 (red) and pSc119.2 (green) clones were used as FISH probes. Yellow arrows denote quadrivalents.
