# Supplementary figures and images for: Chromosomal instability and phenotypic variation in a specific lineage derived from a synthetic allotetraploid wheat
Source: Front Plant Sci. 2022 Aug 22;13:981234. doi: 10.3389/fpls.2022.981234 (PMC9441941; doi:10.3389/fpls.2022.981234)

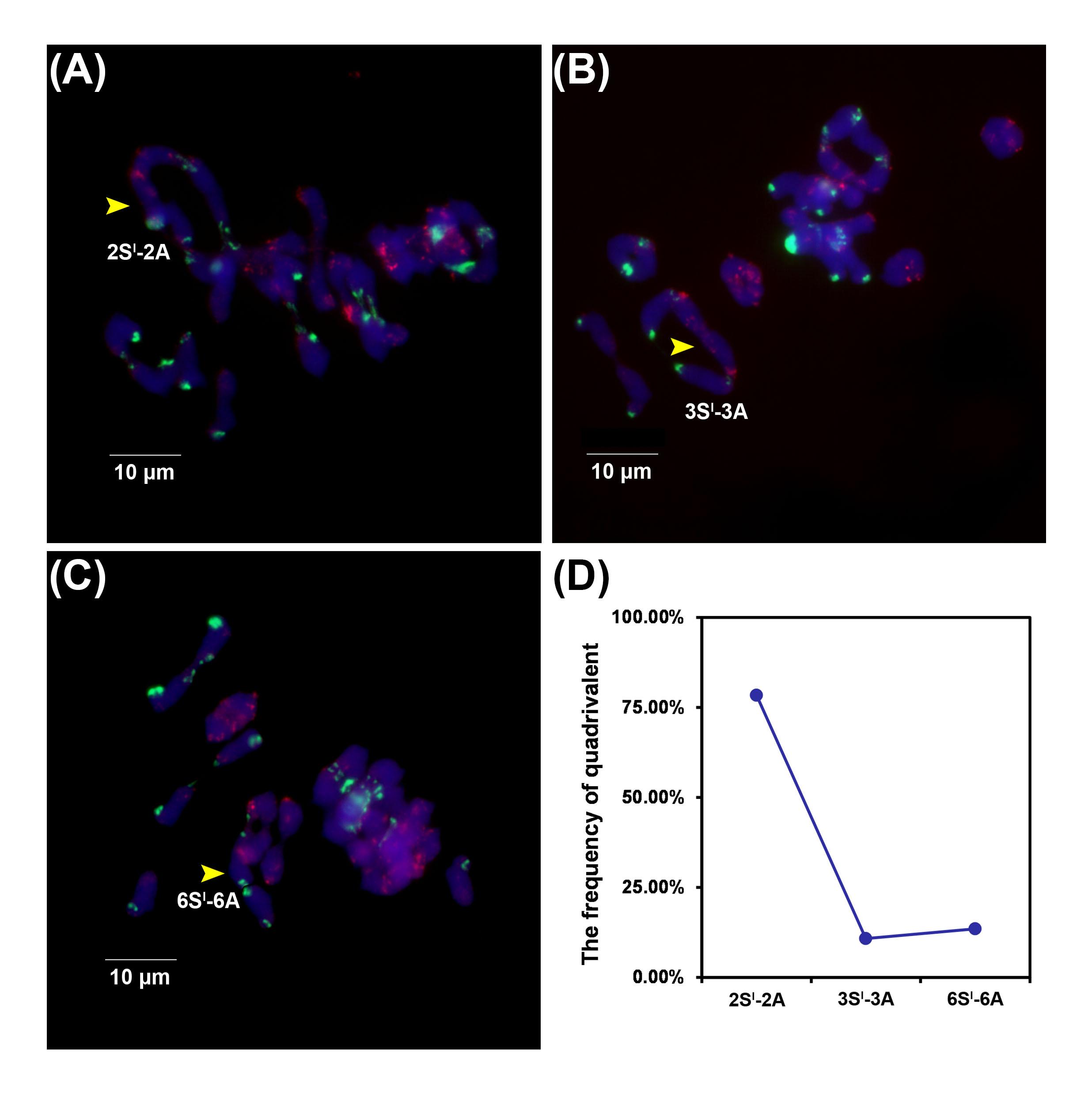

Supplement: Supplementary Figure 1 — Occurrence and distribution frequencies of quadrivalents at metaphase I of pollen mother cells (PMCs) among three homoeologous chromosome pairs in which quadrivalent occurred. These were 2Sl-2A (A), 3Sl-3A (B), and 6Sl-6A (C); their distributions were quantified (D). The pAs1 (red) and pSc119.2 (green) clones were used as FISH probes. Yellow arrows denote quadrivalents. [file Image_1.JPEG]
